# Optimized Detection of Acidic ADP-Ribosylation by PARP14 *in Vitro* Using Multi-Modal Mass Spectrometry Reveals Putative Autoregulatory Patches

**DOI:** 10.1016/j.mcpro.2026.101666

**Published:** 2026-09-21

**Authors:** Steph Nivaggioli, Hannah H. Nguyen, Kiana K. Harker, Christian M. Mohr, Jake Wood, Mikayla Dupen, Zeeshan Javed, Natalia Karjalainen, Violetta Yousefpor, Ashok Reddy, Ian Carter-O’Connell

**Affiliations:** 1 Department of Chemistry and Biochemistry, Santa Clara University, Santa Clara, California, USA; 2 Proteome Shared Resource, Oregon Health and Science University, Portland, Oregon, USA

**Keywords:** ADP-ribosylation, PARPs, PARP14, post-translational modification, proteomics, mass-spectrometry, TLC-MALDI-TOF

## Abstract

ADP-ribosylation is a highly dynamic process that is increasingly linked to a diverse set of cellular functions and pathophysiological outcomes. The modification of target substrates (*e*.*g*., proteins, DNA, RNA) involves ligation to an array of different chemical moieties. Recent work has shown that the ester bonds formed between ribose and aspartate (Asp)/glutamate (Glu) residues are particularly labile. A common strategy to overcome this lability is the replacement of the ADP-ribose (ADPr) by hydroxylamine. Attachment of hydroxamic acid (HA) permanently shifts the mass of labeled Asp/Glu residues by +15 Da. However, this method suffers from two notable limitations: HA treatment for long periods of time will label Asp/Glu sites that are solvent exposed, but not modified by ADPr; and the competing hydrolysis reaction still occurs in the conditions routinely used for HA labeling. Herein, we optimize a MS workflow that overcomes these drawbacks. As a proof-of-principle, a truncated form of PARP14 was assessed for its ability to ADP-ribosylate peptides and itself. Conditions that prioritize HA attack over the background rate of hydrolysis were determined using an ultrathin matrix-assisted laser-desorption/ionization time-of-flight method with ADPr-peptides. These efforts doubled HA labeling efficiency and completely removed ADPr within an hour. Next, a series of MS experiments identified the sites of PARP14 automodification using both standard HA labeling conditions (overnight, pH 7.0, 25 °C) and the optimized conditions. Of the identified 9 ADPr sites on PARP14, five are unique to the optimized conditions and six are newly identified. Mutagenesis revealed a set of sites that down-regulated PARP14 and another set that appears to activate PARP14. Taken together, these efforts have improved the true positive site identification rate using HA and have revealed a novel role for several automodification sites in PARP14 regulation.

## Highlights

• TLC-MALDI optimizes hydroxylamine labeling of acidic ADP-ribosylation sites.
• Optimized MS workflow doubles labeling efficiency for acidic ADPr site detection.
• New MS pipeline identifies nine PARP14 automodification sites, six are novel.
• A cluster of automodification sites in the KH domain downregulates PARP14 activity.

## Graphical Abstract

**Figure:**
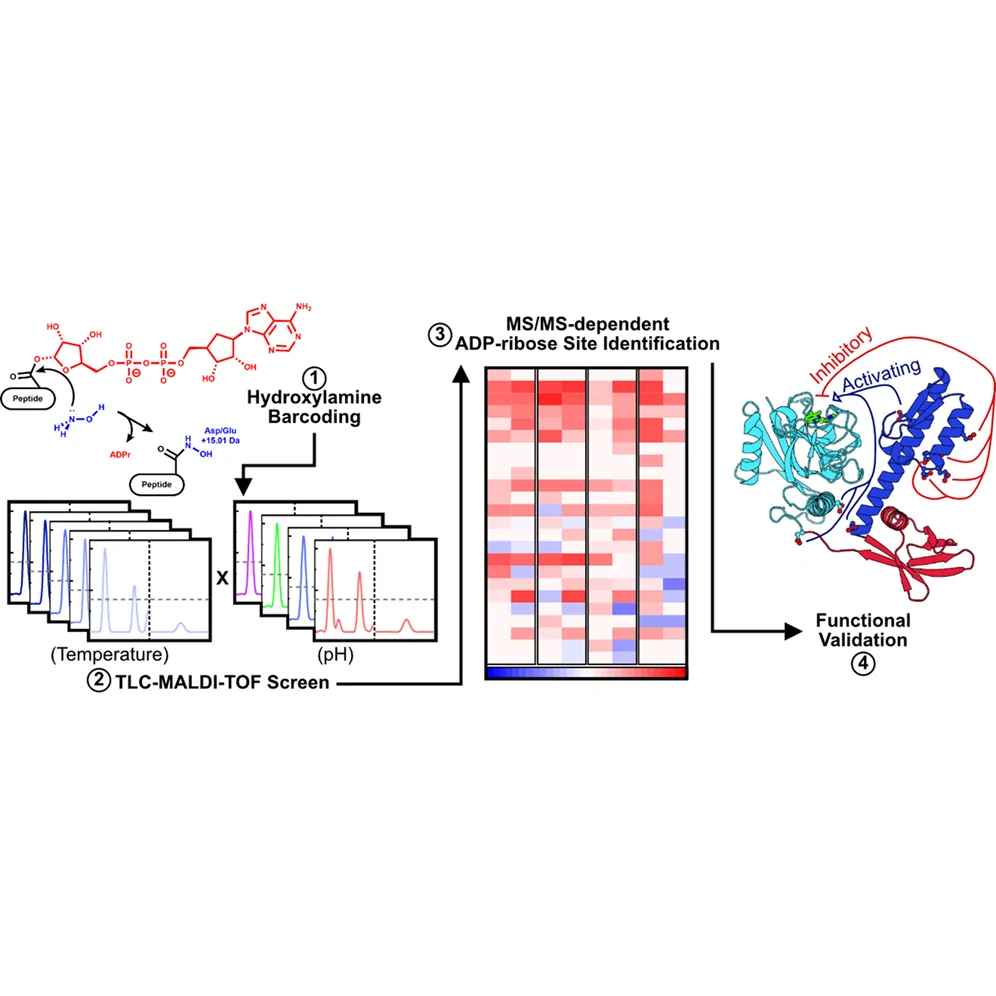

## In Brief

Identifying labile acidic ADP-ribosylation (ADPr) sites on proteins is technically challenging using standard mass spectrometry. By optimizing a hydroxylamine-labeling workflow via TLC-MALDI, we doubled labeling efficiency while significantly reducing false-positive site assignments. Applying this pipeline to the antiviral and cancer-linked enzyme PARP14 revealed nine automodification sites (six novel) and identified a cluster of five residues in the KH domain that functions as a negative autoregulatory patch to tune PARP14 catalytic activity.

ADP-ribosylation (ADPr) is an ancient, highly conserved, reversable post-translational modification (PTM) that is found in all kingdoms of life (1, 2, 3). In humans, the transfer of ADP-ribose onto target macromolecules from the nicotinamide adenine dinucleotide (NAD^+^) substrate is catalyzed by 17 enzymes that comprise the poly-ADP-ribose polymerase (PARP) family (4, 5, 6). The PARP family is divided into three distinct sub-families based on their specific catalytic mechanism (7). PARPs 1, 2, 5a, and 5b catalyze both mono-ADP-ribose (MAR) transfer (where a single ADP-ribose is attached to the target) and poly-ADP-ribose transfer (where additional ADP-ribose units are attached to ADP-ribose itself) (8, 9). Meanwhile, PARPs 3 to 4, 6 to 8, 10 to 12, and 14 to 16 only catalyze MAR transfer (10), while PARPs 9 and 13 do not retain any transfer activity (11). Perhaps the most actively studied role for PARP activity in the cell is the role of PARP1 in DNA break repair (12, 13), which has led to a number of FDA approved chemotherapeutics (14). However, recent efforts have highlighted the role of the PARP family in a wide range of physiological (*e*.*g*., RNA metabolism (15, 16, 17), cellular trafficking and mobility (18, 19, 20), learning and memory (21)) and pathophysiological pathways (*e*.*g*., viral infection (22, 23, 24, 25, 26, 27), cancer (28, 29, 30, 31), neurodegeneration (32), infertility (33)). As with other PTM classes, ADPr is sensed by a variety of proteins with ADP-ribose recognition domains (34, 35, 36, 37)–so called “readers”–and it is reversed by a varied set of glycohydrolases–the ADP-ribose “erasers” (38, 39, 40, 41). The readers and erasers have likewise been the focus of recent studies linking them to their own cellular and disease pathways (40, 42, 43, 44, 45, 46).

An outstanding hurdle in parsing the function and cellular mechanism for the PARP family is linking a specific site of ADPr to its distinct purpose in the cell. Part of the challenge stems from the fact that at least nine different amino acids have been described as ADPr acceptors (Glu, Asp, tyrosine, serine, threonine, cysteine, histidine (His), arginine, and lysine) (10, 47, 48, 49, 50, 51, 52, 53). Even grouping the acceptors based on the shared chemistry of the formed bond (*i*.*e*., esters *versus* ethers *versus* n-glycosides), this represents a highly differential set of linkages that each require a fundamental understanding of their chemical properties to confidently identify. Recently, a series of reports demonstrated that the ester linkage with ADPr (made by Asp/Glu residues) is unstable at pH > 6.5 and at temperatures greater than 4 °C (54, 55, 56). The other linkages appear to be more robust in alkali conditions and at a range of temperatures, but this means that Asp/Glu modifications require additional care when handling (and might be lost completely based on sample treatment). Given the issues with determining the chemistry of ADPr attachment, labs have developed chemical biology techniques to selectively remove ADPr from different amino acids using a variety of reagents (57, 58). For acidic linkages it has been established that incubation of Asp/Glu-ADPr with hydroxylamine results in the selective removal of ADPr from these sites and its replacement with a hydroxamic acid moiety (59). This chemical ‘scarring’ by HA is observed as a +15 Da mass shift wherever an ADPr is removed (Fig. 1*A*) (60). However, despite decades of work with HA, there are scant reports regarding the competition of HA with the background hydrolytic rate of ADPr removal, nor the experimental conditions that would favor HA transfer and limit background labeling with HA in the absence of ADPr (a known complication (61)).

**Fig. 1:**
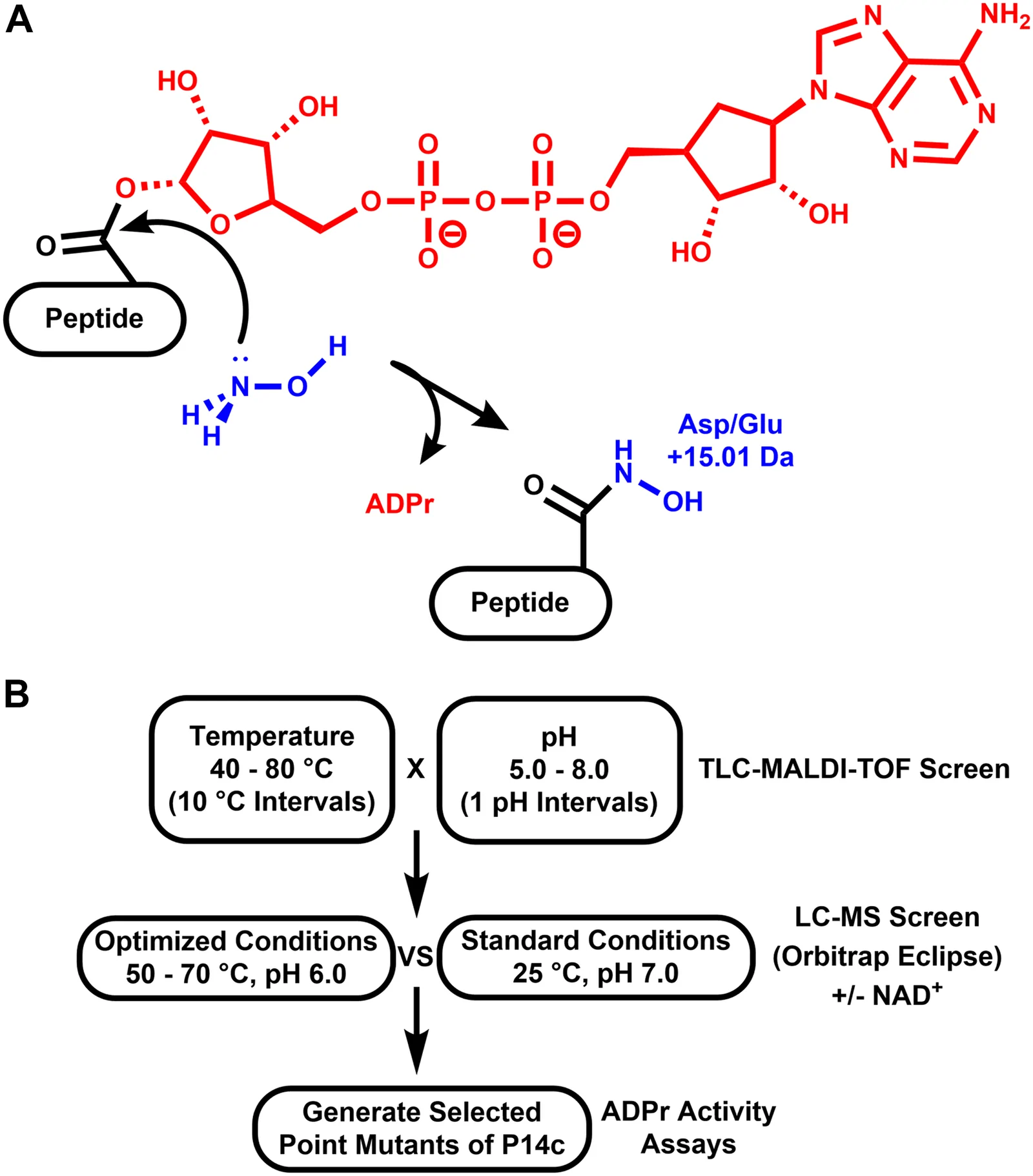
**Overview of the experimental approach.***A*, ADP-ribosylation of peptidyl substrates (step 1) and replacement of ADPr with HA (step 2). *B*, MS workflow and site validation. ADPr, ADP-ribose; HA, hydroxamic acid.

High-resolution MS has been leveraged in the past 15 years to address the problem of ADPr site identification (62). These efforts have contributed to a growing body of thousands of putative ADPr sites (63). However, the lability of the peptide-ADPr linkage and, more importantly, ADPr itself constrains the options for site identification. The most readily accessible tandem mass spectrometry method, higher-energy collisional dissociation (HCD), is not compatible with ADPr site identification as the collisional energy required to generate fragment peptide ions removes ADPr itself (50). HA treatment is an alternative, but it only works with acidic residues (59), and its compatibility with MS pipelines is understudied–especially in light of the recent reports highlighting the fragility of the ester ADPr-peptide bond (54, 55, 56). Electron transfer dissociation (ETD) has been successfully deployed in the past decade as an alternative to HCD for ADPr site identification (47). The softer ionization energy deployed in ETD preserves the ADPr moiety on the fragmented peptide, allowing for site assignment. However, ETD is not nearly as accessible as HCD for most proteomic workflows as there are fewer instruments capable of performing ETD (with manufacturers removing ETD modules from the base models of new instruments), the reagents required for ETD (*e*.*g*., a fluoranthene source) increase sample costs and overhead, and there is a barrier to entry given the technical knowledge required to successfully implement ETD as compared to HCD (64, 65, 66). Regardless of the method chosen, there is a clear need for optimized MS pipelines to address the differential chemistries of ADPr linkages and for additional protocols to decrease false negative discovery rates due to ADPr loss in peptide library preparation.

While the role of PARP1-, and 2-mediated ADPr, and its downstream impacts on DNA repair has been extensively studied (67, 68), the function of ADPr signaling outside of this canonical role remains poorly understood. A subset of PARPs that have been the intense focus of current research efforts, and continue to surprise with novel mechanistic questions, are the interferon-inducible, antiviral PARPs (PARPs 7, 9–14). These PARPs are characterized by a set of putative RNA binding domains found toward their N-terminus and a highly conserved MAR catalytic domain at the C-terminus (with the notable exceptions of PARPs 9 and 13, which lack catalytic activity) (7). Evidence of positive evolutionary selection in response to viral-based challenges within these antiviral PARPs is indicative of a robust and dynamic host-pathogen arms race (23, 69, 70). Perhaps unsurprisingly, many of these antiviral PARPs are linked to immunity processes and inflammatory signaling (71, 72).

Within this sub-family of antiviral PARPs, PARP14 is emerging as a versatile and multi-faceted enzyme (recent reviews (73, 74, 75) have highlighted its varied cellular roles). As the largest of the human PARPs, PARP14 is 1801 amino acids long and contains several functionally distinct domains (7) (most of which remain poorly characterized). In addition to several putative RNA-binding domains at the N-terminus, PARP14 has 8 KH domains, an ADPr glycohydrolase domain, three ADPr binding domains, and the ADPr transfer domain. As with the other antiviral PARPs, PARP14 is an important modulator of the innate antiviral response (76) and inflammatory signaling (77). PARP14 is a key factor in cancer progression in several cancer types (notably, multiple myeloma (29) and hepatocellular carcinoma (28)) and is linked to cancer immunity regulation (78). Recently, PARP14 was shown to have a significant antiviral role during coronavirus infection (79). PARP14 is massively upregulated in response to interferon stimulation and is thought to catalyze the majority of MAR transfer in these conditions (22, 80, 81). Beyond these immunity-linked functions, PARP14 plays key roles in supporting genome stability through interactions with the DNA break sensor KU complex and the MRE11 nuclease (82). Loss of PARP14 has been shown to increase DNA replication stress and homologous recombination repair defects in cells (75, 83), though the direct links between PARP14-dependent ADPr and genome stability maintenance remain unknown. Further, PARP14 modifies several proteins that are involved in the DNA damage response and it is implicated as a regulator of RPA complex function (41) (a known single-stranded DNA-binding factor involved in DNA repair (84)). Additionally, PARP14’s interaction with RACK1 in ovarian cancer cells is implicated in stress-granule formation and maintenance (85). This PARP14-RACK1 interaction was shown to support tumor cell adaptation to stress and may play important roles in normal and pathological stress-granule function. Moreover, PARP14 plays a role in cell-cycle progression through stabilization of cyclin D1 mRNA (86). Recently, several labs have independently shown that PARP14 activity in the cell is modulated by the PARP9/DTX3L complex (80, 87, 88) and these three proteins are implicated in cross-PTM regulation with ubiquitin (80, 89, 90, 91, 92). In short, while PARP14 was initially identified based on its role in immune processes, it is integrated in a diverse set of cellular pathways. PARP14’s full characterization requires an investigation of both its binding partners and its MARylation targets.

Like many other PARP family members, PARP14 undergoes significant automodification upon activation (80, 93, 94), though the function of this auto-MARylation remains poorly understood. PARP14 attaches MAR onto target proteins primarily though Asp/Glu residues (54, 93), making it compatible with HA-based approaches. As noted above, PARP14 possess distinct domains that can write, read, and erase ADPr within a single polypeptide (7). This arrangement of activities and intermolecular interactions increases the complexity of assigning function to each modification catalyzed by PARP14, as it is likely acting in a highly dynamic manner. Fortunately, there are multiple truncations of PARP14 that have been validated in the literature that simplify the intramolecular editing functions by removing the MAR reader/editor domains. In particular, the PARP14 truncation that only contains the three C-terminal domains (the eighth KH domain, a WWE domain, and the catalytically active ART domain) has been extensively utilized *in vitro* (54, 80, 94, 95, 96). As a proof of principle, we focused our efforts on this PARP14 variant (PARP14c) to limit potential complications and allow us to optimize our MS workflow. Taken together, PARP14c thus represents an intriguing and promising target for linking specific ADPr modifications to downstream effects.

An additional challenge towards optimizing MS-based workflows is the cost and time required to test a wide-array of conditions quickly. Ultrathin matrix-assisted laser-desorption/ionization time-of-flight (TLC-MALDI) represents an ideal technology to overcome these limitations as it can be completed in under an hour (97), is compatible with direct peptide labeling and stability assays (54, 94), and can be performed with buffer solutions not normally compatible with traditional MALDI-TOF (98)–that can then be deployed in proteomics workflows directly. We previously demonstrated that PARP14-dependent modification is compatible with TLC-MALDI and we isolated a 5-mer minimal motif that is selectively ADP-ribosylated by PARP14 on Asp/Glu (54). Herein, we deploy TLC-MALDI with this ADPr-peptide to rapidly screen a spectrum of temperatures and pHs to determine their effects on HA attachment (Fig. 1*B*). We prepared a series of peptide libraries of auto-MARylated PARP14c for HCD analysis, allowing for the direct comparison of our optimized conditions with those routinely employed for MAR site analysis. Using these methods, we were able to nearly double the number of identified ADPr sites compared to traditional HA sample preparations. Further, we significantly decreased the false-positive discovery rate while using HA as a reagent with a simple control. Mutation analysis of the identified sites revealed a cluster of five residues that negatively downregulate PARP14c activity *in vitro*. Interestingly, loss of any single site had no effect on PARP14c activity, suggesting that the patch of residues regulates PARP14, not one specific site. Taken together, our results optimize a technique being employed in our field for the identification of putative ADPr modification sites and we have used these data to identify a negative regulatory motif on PARP14.

## Methods

### Experimental Design and Statistical Rationale

PARP14c was selected based on its robust automodification both *in vitro* and *in vivo* and the observation that the bulk of this activity is localized to acidic residues (54, 55, 56, 93, 94). The peptide used for MARylation was previously identified as a robust PARP14 target (54) and was originally identified as a *bona fide* target of PARP14 MARylation on PARP13 (93, 94). All peptide MARylation assays were completed in triplicate with separate aliquots of recombinant PARP14c and unmodified peptide. All PARP14c ADP-ribosylations were completed in triplicate with separate aliquots of PARP14c. This number of replicates provides enough data for outlier detection and enables standard statistical analysis using Student’s *t*-tests. Student’s *t*-tests were performed with a two-tailed distribution to determine significance for any observed differences between experimental conditions in Excel Professional Plus 2016 (Microsoft, Redmond, WA). All tandem MS experiments were completed in duplicate with separate aliquots of recombinant PARP14c. Given the depth of MS sequencing with a single known target, the use of biological duplicates provides sufficient sequencing coverage to determine correlation between identified sites between independent replicates. Specific controls used for each assay are described below in the associated methodological section.

### Recombinant Proteins and Peptides

Recombinant proteins were preceded by a 6x His tag in a pET-28b(+) vector and were expressed and purified as previously described (99). PARP14 was derived from a human variant and its Uniprot identifier is Q460N5. PARP14c comprises the residues from 1459 to 1801. Bovine serum albumin BSA was commercially sourced (Bio-rad, Hercules, CA). Mutant variations of PARP14c were designed *in silico* to convert targeted Asp/Glu residues to Ala. Codon-optimized synthetic DNA gene fragments (Integrated DNA Technologies, Coralville, IA) containing these mutated sites were amplified by PCR using custom DNA oligonucleotides (Integrated DNA Technologies) and were inserted into the WT PARP14c sequence using restriction enzyme cloning. Expression vectors were sequenced to confirm DNA insertions and mutations. Protein concentrations were determined using a gel-based assay with BSA as a standard. A representative Coomassie stained gel used for protein determination loaded with all PARP14c variants can be found in Supplemental Fig. S2. The peptide used for MARylation was commercially synthesized (GenScript, Piscataway, NJ). Its sequence is: HREISW. Chemoenzymatic synthesis and purification of the MAR-peptide was completed as previously described (54).

### Hydroxylamine Peptide Assay

Synthesized MAR-peptide was equilibrated to 50 μM in 10 mM potassium phosphate, pH 6.0 and diluted in 1 M hydroxylamine (RICCA Chemical Company, Arlington, TX), pH 5.0, 6.0, 7.0, or 8.0 to a final concentration of 10 μM. MAR-peptides (2 μl) were incubated at either 25, 40, 50, 60, 70, or 80 °C and samples were quenched at 1 h in 18 μl of 0.1% TFA. 2 μl of sample was mixed with 4 μl of TLC-MALDI buffer (MB: 5 mg/ml α-cyano-4-hydroxycinnamic acid (CHCA, Sigma-Aldrich, St Louis, MO), 25% acetonitrile, 0.05% TFA, and 5 mM NH_3_PO_4_) and subjected to TLC-MALDI analysis.

### TLC-MALDI Preparation and Cleanup

The ultrathin layer was prepared as previously described (94). Briefly, CHCA was dissolved in acetone to a concentration of 20 mg/ml. A 10 μl pipette tip was filled with the CHCA-acetone saturated solution using capillary action and a thin-layer of matrix was deposited on the MALDI objective followed by rapid evaporation of the volatile solvent. 2 μl of sample in MB was spotted directly on the thin-layer and dried. After drying, the spots were washed twice with 5 μl of ice-cold 0.1% TFA for 5 min. Calibrations were performed using Peptide Calibration Standard II (Bruker, Billerica, MA) per the manufacturer’s instructions. MALDI-TOF experiments were performed using a MALDI-8020 instrument (Shimadzu, Kyoto, Japan).

### TLC-MALDI Acquisition Parameters and Data Analysis

The collection of peptide spectra was performed using an automated firing method defined by a regular circle with a TV Raster (1500 μm diameter, 50 points, no offsets, 199 μm spacing) and random dithering (250 ms dwell time, 20 μm radius) with a mass range set to 700 to 1500 Da. Fifty laser shots were fired for each profile at a frequency of 50 Hz, and 2500 total shots were collected per sample. Post-acquisition baseline subtraction and smoothing were performed using MALDI Solutions (Shimadzu) with the following parameters: baseline filter width set between 15 to 30, Savitsky–Golay smoothing with a smoothing width between 5 to 30, and peak width set to 5. The peak delimiter method was set to threshold apex with a threshold of 0.01 mV and exclusion of peaks with intensities less than 0.01 mV. Following data acquisition, selected unmodified, HA-modified, MAR-modified, and HA-MAR-modified peaks were identified based on their expected mass and integrated to determine the area under the curve using MALDI solutions. The resulting values were used to calculate relative levels of HA modification. The peptide modification ratio was calculated by dividing the area from the HA-modified peak by the sum of the unmodified, HA-modified, MAR-modified, and HA-MAR-modified peak. The center of each peak was recorded, and the spectra were rejected if any observed mass differed from the predicted mass by more than 1%. The spectral intensities were min-maxed normalized between 0.0 and 1.0 in Origin 2020 (OriginLab, Northampton, MA) and centered on the unmodified peak to aid comparisons between replicates.

### PARP14c MS Sample Preparation

The PARP14c MAR site identification experiments were performed with 33 μg of recombinant PARP14c in a 175 μl reaction volume consisting of 50 mM Hepes, pH 7.5, 100 mM NaCl, 12 mM MgCl_2_, and 0.5 mM TCEP that was labeled for 30 min at 30 °C with 100 μM NAD^+^ (VWR, Radnor, PA). Mock reactions were performed in identical conditions, except without the addition of NAD^+^. ADPr was quenched by the addition of 200 μM PJ-34 (Tocris Bioscience, Abingdon) and the PARP14c samples were diluted 1:1 in 2 M hydroxylamine, pH 6.0 or pH 7.0. The samples equilibrated at pH 7.0 were held at 37 °C for 12 h while the samples at pH 6.0 were held at either 50, 60, or 70 °C for 1 h. Mock samples were similarly equilibrated at pH 7.0 and 37 °C for 12 h (standard conditions) or at pH 6.0 and 70 °C for 1 h (optimized conditions, highest temperature used for background HA labeling rate). MARylated PARP14c was digested via proteolysis with 50 μl of 20 ng/μl trypsin (Promega, Madison, WI) for 12 h at RT and purified using filter aided sample prep as previously described (48).

### LC-MS Instrumentation and Analysis

Proteolyzed samples were dried via speedvac and reconstituted in 5% formic acid at ∼1ug/ul. 2ug/sample was injected into a Dionex RSLC UHPLC (Thermo Fisher Scientific, Waltham, MA). Peptides were delivered to an Acclaim PepMap 100 μm × 2 cm NanoViper C18, 5 μm trap (Thermo Fisher Scientific) on a switching valve at 5ul/min. After 5 min of loading, the trap column was switched on-line to a PepMap RSLC C18, 2 μm, 75 μm × 25 cm EasySpray column (Thermo Fisher Scientific). Peptides were then separated at low pH using a 5 to 30% ACN gradient over 60 min in a mobile phase containing 0.1% formic acid at 300 nl/min flow rate. Tandem mass spectrometry data was collected using an Orbitrap Eclipse Tribrid instrument configured with an EasySpray Source (Thermo Fisher Scientific). MS1 survey scans were performed in the Orbitrap mass analyzer (resolution = 120,000), and data-dependent MS2 scans performed in the linear ion trap using HCD fragmentation (normalized collision energy = 35) following isolation with the instrument’s quadrupole with isolation width set at 1.6 m/z.

Raw MS scans were analyzed with the Byonic suite of algorithms (v4.2.10, Protein Metrics, Boston, MA) (100, 101). The data were searched against a database containing His-PARP14c and the *Escherichia coli* proteome (Uniprot: UP000002032, June 2009, 4156 sequences), as well as decoys and contaminants included as a default in Byonic. Complete lists of identified proteins are collected in Supplemental Table S1. Preliminary analysis was performed using PMi-Preview (v4.2.10) to establish the optimal precursor mass tolerance and fragment mass tolerance for each MS/MS data file. The following parameters were used for the final analysis performed with PMi-Byonic (v4.2.10): initial precursor mass tolerance (4–6 ppm) and fragment mass tolerance (0.3–0.6 Da) set by PMi-Preview; recalibration of fragment and precursor mass from PMi-Preview; fully specific digestion with up to two missed cleavages. Variable modifications included oxidation (M) and hydroxamic acid (DE) with a static modification of carbamidomethyl (C). Up to two HA modifications were allowed for each peptide ID. False discovery rate was set to 1% for both peptides and proteins. For the sites we considered as *bona fide* MAR sites we required that the peptide fragments have a Byonic peptide score greater than 300 (considered a “good” match by the Byonic algorithm), a PEP-2D score equal to or less than 0.01 (consistent with an false discovery rate of 1% for PTM localization), and an enrichment over background (without NAD^+^) ≥ 2.5 in both replicates (to eliminate potential false positives), and we computationally validated the representative spectra using Byonic. Complete lists of identified HA sites are collected in Supplemental Table S2. To avoid infinite or undefined log_2_ fold changes when a spectral count was zero in one condition, a pseudocount of 0.5 was added to all spectral counts prior to computing log_2_ enrichment values (log_2_((sample + 0.5)/(background + 0.5))). This approach follows established spectral-count proteomics practice (102) and has been used in recent proteomics studies (103). Using 0.5 *versus* 1.0 as the pseudocount did not materially change the ranking of top enriched proteins (Spearman ρ = 0.998, *p*-value <0.001) indicating robustness of the chosen small constant. Log_2_ enrichment scores are collected in Supplemental Table S3. Pearson coefficients were calculated using standard statistical methods and are collected in Supplemental Table S4.

### PARP14 ADP-Ribosylation Assays

PARP14c (2.75 μM; WT, 9 AM, Pa, KHH, Hi, or the single site mutants) was incubated with 100 μM NAD+ for 15 or 60 min at 20 °C in an 18 μl reaction volume consisting of 50 mM Hepes, pH 7.5, 50 mM NaCl, 12 mM MgCl_2_, and 0.5 mM TCEP. Substrate labeling was detected via immunoblot using the mono-ADP-ribose antibody AbD33204 (Bio-rad) and quantified using ImageLab v5.2 (Bio-Rad). The ADPr activity was normalized against the most active PARP14c variant’s activity at 60 min. ADPr assays with BSA were performed as above, except with 200 nM PARP14c and 1.25 μM BSA. Substrate labeling was min-maxed normalized between 0.0 and 1.0 in Origin 2020 (OriginLab).

### Publicly Available PARP14c Sequences and Sequence Alignment

Based on prior phylogenetic analysis of primate (69) and metazoan (104) PARP14 proteins, nine representative primate species and five representative non-primate metazoan species were selected for multi-sequence alignment. The publicly available proteomes from human (*Homo sapiens*), chimpanzee (*Pan troglodytes*), gorilla (*Gorilla gorilla*), orangutan (*Pongo abelii*), white-cheeked gibbon (*Nomascus leucogenys*), rhesus macaque (*Macaca mulatta*), baboon (*Papio anubis*), marmoset (*Callithrix jacchus*), mouse (*Mus musculus*), chicken (*Gallus gallus*), lizard (*Anolis carolinensis*), frog (*Xenopus tropicalis*), and fish (*Danio rerio*) were queried against the PARP14c protein sequence using NCBI-BLAST v2.17.0 (105). The species-specific PARP14 sequence with the highest e-value and identity score was chosen for alignment and the sequence were trimmed to match the length of PARP14c. Sequences were arranged based on previous phylogenetic trees (69, 104) and the multi-sequence alignment was completed using default settings in CLUSTAL Omega v1.2.4 (106).

## Results

Initially, we set out to adapt our previous work with TLC-MALDI (54, 94) to screen sample preparation conditions that prioritize labeling by HA over the background hydrolytic rate. We also wanted to significantly shorten the traditional HA labeling time (12–24 h) as we hypothesized that a longer incubation increases the risk of ADPr hydrolysis without attendant HA modification (54, 55). Further, longer incubation may increase the rate of background HA labeling on unmodified acidic residues (61). TLC-MALDI involves the preparation of an ultrathin matrix layer directly on the MALDI objective and the formation of matrix-analyte seed crystals directly on this surface (97, 98). Due to the hydrophobic properties of the αCHCA matrix, additional wash steps with cold TFA will remove chemicals that are required for catalysis but are not compatible with MALDI-TOF analysis (*e*.*g*., NaCl, buffer salts, detergents, etc.). Most of the proteomic workflows used to identify MAR (or to perform proteolysis of any sort) involve a long incubation (47, 58, 60, 93, 99, 107, 108, 109, 110)–usually overnight–with protease (primarily trypsin) at RT or 37 °C in buffers equilibrated to pH 7.5 to 8.5. As expected, incubating the labeled peptide in mild conditions (pH 7.0) in the absence of HA at 40 °C results in significant hydrolysis of ADPr over the course of one hour (Fig. 2*A*). Addition of HA results in the complete loss of ADPr and the appearance of a new +15 Da HA-peptide peak. Interestingly, even in the presence of an excess of HA, the rate of conversion to the HA-peptide is only ∼30%. These data indicate that previous efforts at HA labeling (60, 107, 109, 110, 111) were potentially massively undercounting ADPr site modifications on acidic residues. Decreasing the pH to 6.0 results in the appearance of a minor HA-ADPr-peptide peak (at +556 Da) and a similar +15 Da HA-peptide peak as in the pH 7.0 conditions.

**Fig. 2:**
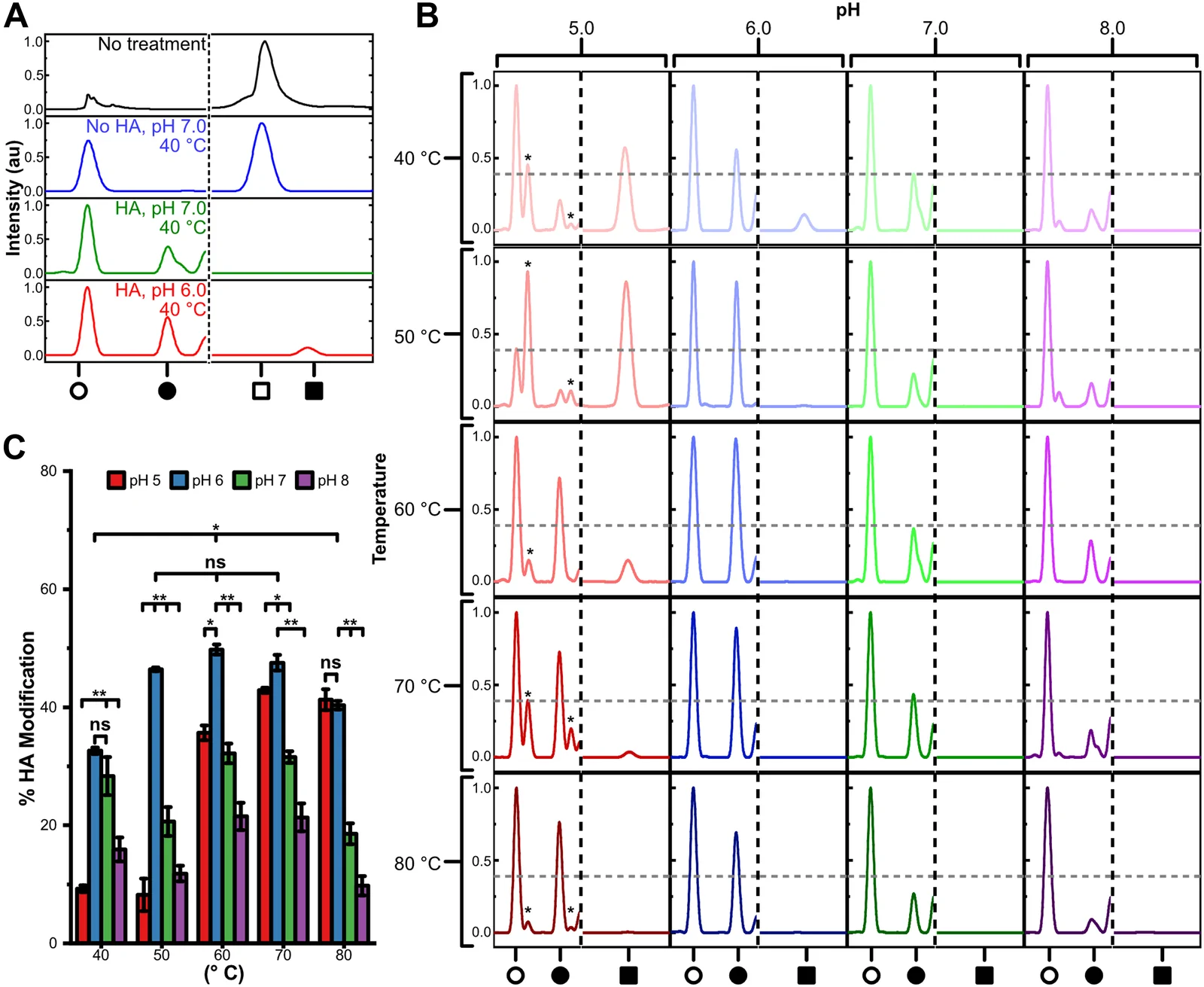
**Optimization of conditions that favor HA transfer using TLC-MALDI.***A*, a synthesized MAR-peptide (*black*) was equilibrated at 40 °C and pH 7.0 (*blue*), and MAR hydrolysis was monitored at 1 h using TLC-MALDI. The mass peaks associated with either modified or unmodified peptide are shown for comparison. To determine the extent of HA transfer, the same MAR-peptide was equilibrated in the presence of 1 M hydroxylamine at 40 °C and pH 7.0 (*green*) or pH 6.0 (*red*) for 1 h ○: unmodified peptide, •: HA-peptide, □: MAR-peptide, ▪: HA-MAR-peptide. *B*, HA transfer was systemically investigated using TLC-MALDI at temperatures between 40 to 80 °C (y-axis) and pHs between 5.0 to 7.0 (x-axis) as in (*A*). The *dashed horizontal line* indicates the intensity of the HA-peptide peak observed at 40 °C and pH 7.0 (standard conditions). At pH 5.0 peak splitting is indicated by an ∗. *C*, MS spectra were integrated to determine the relative levels of HA modification. The bar graphs depict the fraction of total peptide that was modified by HA (mean ± S.E.M., n = 3). ∗ represents *p*-value <0.05, ∗∗ represents *p*-value <0.01, two tailed Student’s *t* test, n.s. represents a nonsignificant difference. HA, hydroxamic acid; MAR, mono-ADP-ribose.

From these initial data, we established a baseline for the amount of HA labeling that occurs under the preparation conditions routinely utilized for proteomics. Given our previous work measuring the kinetics of ADPr hydrolysis using TLC-MALDI, we wanted to determine if temperature and/or pH would have a differential effect on the rates of hydrolysis *versus* nucleophilic attack by HA on the ester bond. As our previous work determined that the optimal pH to stabilize the ADPr modification is 6.0 (54), we chose a range of buffers equilibrated to pHs between 5.0 to 8.0. We hypothesized that limiting the rate of hydrolysis under acidic conditions would have a beneficial effect on HA attack. However, the decrease in pH could deactivate the nucleophile (HA), so it’s essential to find an optimum pH to lower the hydrolytic rate while not slowing the rate of HA attack. Similarly, as our initial data indicated that temperatures above 80 °C favor hydrolysis for all pHs tested, we picked a range of temperatures from 40 to 80 °C for evaluation in 10 °C increments. We knew from our previous work that temperatures below 40 °C significantly slow the rate of hydrolysis (54), but they would also significantly slow the rate of HA attachment. Colder conditions would therefore require longer incubation steps – which we want to avoid during peptide library generation as detailed above.

As with our preliminary experiments at 40 °C, there was a negligible difference between the amounts of HA modification at the various pHs tested, with the noted decrease in labeling under alkali conditions (Fig. 2*B*). We do observe some peak splitting at pH 5.0 that was not observed at any of the other pHs. The doublet observed at pH 5.0 likely arises from coexisting ion populations produced during the MALDI process. MALDI mass spectra are known to be highly sensitive to matrix/sample solution composition and pH, which affect co-crystallization and ionization equilibria (112). For this pH we integrated the area under the split peaks for all subsequent analysis. Increasing the temperature by 10 °C resulted in a significant increase in HA labeling at pH 6.0 (Fig. 2*B*). All of the other tested pHs resulted in either a decrease or no change in HA labeling efficiency. As temperature is elevated, there is an insignificant increase in labeling at pH 6.0, and a significant increase in labeling with the other pHs. At 80 °C, there is a diminishing return on HA labeling at all pHs tested. Taken together, our data indicate that the optimal conditions to preferentially scar acidic MAR sites with HA are in buffers equilibrated to a pH of 6.0 and incubated between 50 to 70 °C. These two simple changes in method resulted in a nearly two-fold increase in HA labeling (28.3% labelling *versus* 49.8%) compared to the conditions routinely used for HA scarring (pH 7.0, 25–37 °C, overnight incubation).

Next, we wanted to determine what impact our optimized conditions would have on MS/MS based site discovery. Given our previous work with the PARP14c construct *in vitro*, it’s emerging roles in innate immunity and cancer progression, and the scant information regarding its autoregulation, we settled on this enzyme as our focus for HA optimization. Following ADPr by addition of NAD^+^, recombinant PARP14c was subjected to HA labeling at a pH of 6.0 for either one hour at 50, 60, or 70 °C (optimized conditions) or overnight at room temperature at a pH of 7.0 (standard conditions), preceded by trypsin proteolysis and HCD analysis by LC-MS. Untreated PARP14c was similarly subjected to MS analysis to determine the false positive discovery rate due to background HA labeling on acidic residues (61). Each of the tested conditions was performed in duplicate and we observed 24 sites of ADPr modification at least once in the tested conditions (Fig. 3*A* and Supplemental Tables S2 and S3).

**Fig. 3:**
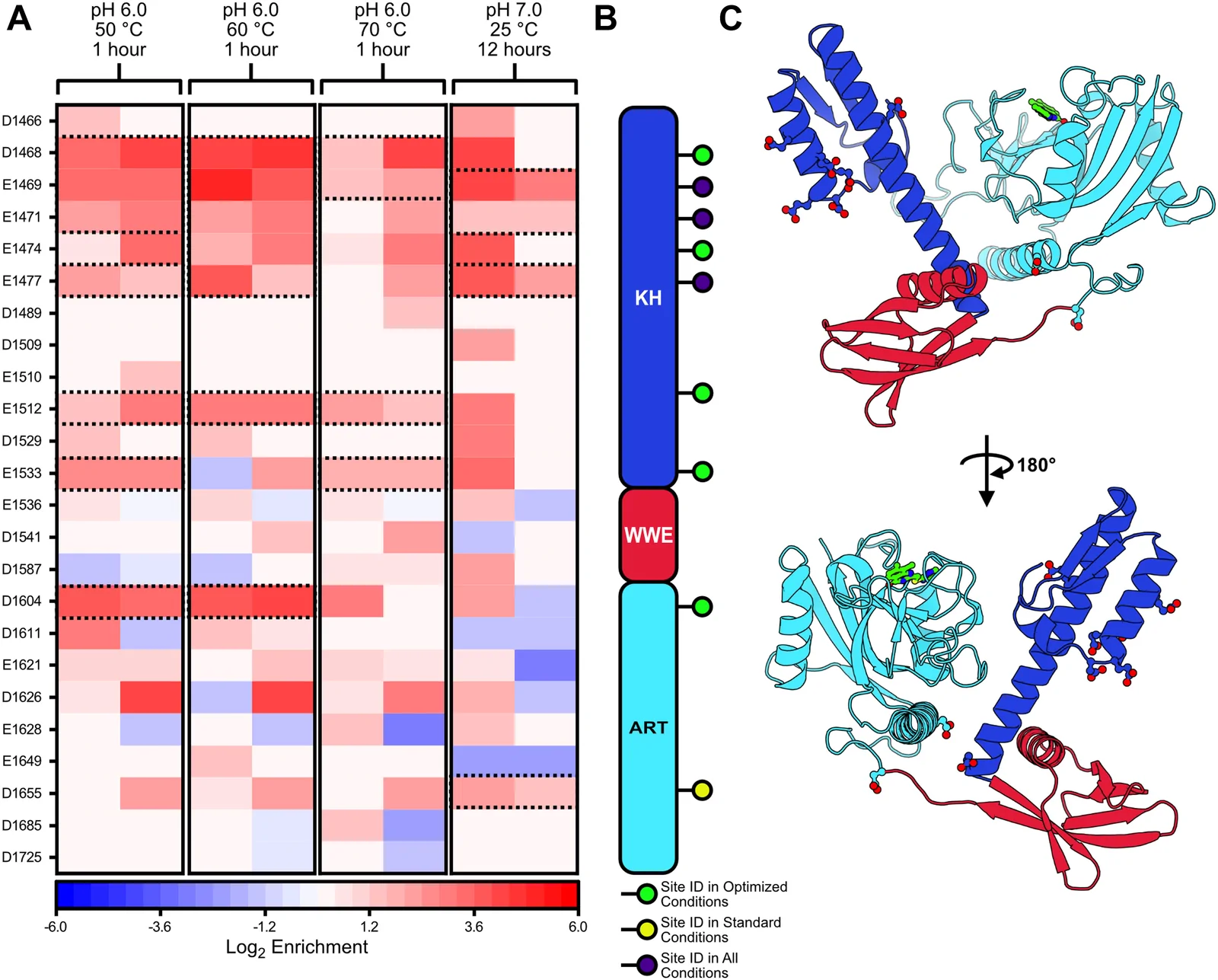
**Differential effects of hydroxylamine incubation conditions on PARP14c MAR site identification.***A*, heat map depicting the fold enrichment (log_2_) of PARP14c sites modified by HA from NAD^+^ treatment *versus* non-NAD^+^ treatment (*background*). Duplicates are grouped by hydroxylamine conditions (*indicated above*) and the specific residue (hPARP14 numbering) is indicated. *Red* corresponds to enrichment in site identification compared to background while *blue* corresponds to a diminishment. Bound *boxes highlight* PARP14c sites that were identified in duplicate with an enrichment ≥2.5-fold. *B*, domain architecture of PARP14c from N-terminus (*top*) to C-terminus (*bottom*). The KH (*blue*), WWE (*red*), and ART (*cyan*) domains are indicated. Sites identified in (*A*) are indicated by filled *circles*. *Purple* indicates sites found in both optimized and standard conditions, *neon green* indicates sites only found in the optimized conditions, and *yellow* indicates sites only found in the standard conditions. Exact residues match the position in the heat map from (*A*). *C*, modelling the MAR sites on the proposed structure of PARP14c. The structure of PARP14 from AlphaFold 2 (125, 126) (Q5460N5 version 4) was aligned with the PARP14 ART domain in complex with the ITK1 inhibitor (6FYM (127), inhibitor indicated in green) using 3D Protein Imager (128) and the nine sites are indicated by sticks. PARP14c domains are colored as in (*B*). HA, hydroxamic acid; MAR, mono-ADP-ribose.

Interestingly, while there was a positive correlation between replicates (Pearson correlation coefficients ≥0.31 for all conditions, Supplemental Table S4), there was variance between peptide counts for all of the tested conditions. The lowest correlation we observed between replicates was for the overnight treatment following MARylation. This could be an indication that the longer incubation at room temperature under mild conditions results in more off-target HA labeling or an increased chance of ADPr hydrolysis, both of which would add to the difficulty in assigning *bona fide* sites in these conditions. Importantly, the inclusion of negative controls where NAD^+^ is not included are essential, as there are clearly hotspots for background HA labeling. This background must be taken into account to remove false positive identifications. As expected, longer incubation results in an increase in false site identifications (90 *versus* 38), so care should be taken to limit HA exposure. In light of these results, we set a requirement that the site be represented with a greater than 2.5-fold enrichment (log_2_) over background to remove potential false positives. When we required that both replicates within a condition pass these screening parameters the list of sites shrunk from 24 to nine. Of those nine, five were only found in the TLC-MALDI optimized conditions, while three were found in both the optimized conditions and the standard methods, and one was found only in the standard conditions (Fig. 3*B*). Of all conditions tested, the one-hour incubation at either 60 °C or 70 °C generated the most site identifications (seven each) and those at 70 °C or under standard conditions produced the fewest (four). The nine sites form three distinct clusters on the structure of PARP14c, with a patch of five centered in the eighth KH domain, two that are located on an elongated helix, and two that are found at the interface of the three domains (ART-WWE-KH) (Fig. 3*C*). Our results indicate that a shorter incubation with HA in slightly acidic conditions (pH 6.0) at temperatures between 50 to 60 °C, can significantly increase the number of true positive identifications while lowering the background HA labeling rate.

Since we optimized a methodology to increase the true positive discovery rate of labile acidic ADPr linkages, we wanted to compare our dataset to prior attempts to identify Asp/Glu-ADPr sites–with a focus on PARP14 automodification sites. We chose studies that facilitated acidic linkages: two of which used the standard HA preparation method (60, 109), two of which utilized ETD as an alternative to HA scarring (56, 113), one of which used a hybrid HCD-electron transfer/higher energy collision dissociation strategy (81), and one of which used neither (114). Across these six studies, only the most recent report by Buch-Larsen and colleagues (56)–which was focused on using sample handling techniques to preserve the labile ester bond and focused on endogenous ADPr site identification–showed overlap with the PARP14 sites we identify herein (Supplemental Table S5). Of those, four are found in both the prior work and ours, three are found in the prior work and not ours, and 20 were only identified herein. Limiting the dataset to the nine high confidence sites reveals an overlap of three sites, with six sites only found in this study. It is worth noting that the prior studies were all focused on the identification of ADPr sites from cell lysates, while our method was focused on an in-depth, *in vitro*, examination of PARP14 automodification. As such, there is most likely dynamic hydrolysis occurring within the cell that could result in the loss of potential sites in that context. Regardless, our work demonstrates a broad promiscuity of PARP14 activity and is indicative of a lack of site preference.

To validate the residues we identified *via* our MS screen as potential MAR sites, we generated a variant of PARP14c where all 9 Asp/Glu residues were mutated to Ala (9 AM). We hypothesized that the loss of these preferential sites would result in an overall lowering of ADPr transfer, as those sites would no longer be able to accept MAR. We grant that the alteration of nine acidic residues to alanine might have an impact on the overall charge and structure of the protein, but the retention of catalytic activity can be used as a coarse-grained proxy for proper folding and enzyme function. Surprisingly, the 9 AM variant was automodified to a greater extent than the WT variant at both short and long intervals (Fig. 4*A*). Our results with PARP14 mirror previous observations that the loss of ADPr ribose sites on PARP1 leads to an apparent increase in automodification at new sites (115). At 15 min there was a 2.2-fold increase in auto-labeling with 9 AM, which decreased to a still significant 1.6-fold change at 60 min (Fig. 4*B*). As the target of modification was the enzyme catalyzing the reaction in these experiments, we were forced to use an enzyme concentration far greater than that normally used in kinetics to observe MARylation using a Western blot. Additionally, we were curious if the increased activity we observed would also affect its *trans*-modification activity. To address this question, we tested the ability of PARP14c to modify BSA at much lower concentrations of PARP14c. Although serum albumin is not a physiological target of PARP14, we hypothesized that the enhanced automodification of the 9 AM mutant may also translate into a significant increase in ADPr of BSA. Indeed, WT PARP14c barely modifies BSA over the course of 30 min (Fig. 4*C*), while incubation with the 9 AM mutant resulted in a 2.3-fold increase in MAR labeling of BSA (Fig. 4, *C* and *D*). Taken together, our results demonstrate that the sites identified by our MS pipeline have a regulatory effect on PARP14c and the loss of those sites constitutively increases PARP14c activity.

**Fig. 4:**
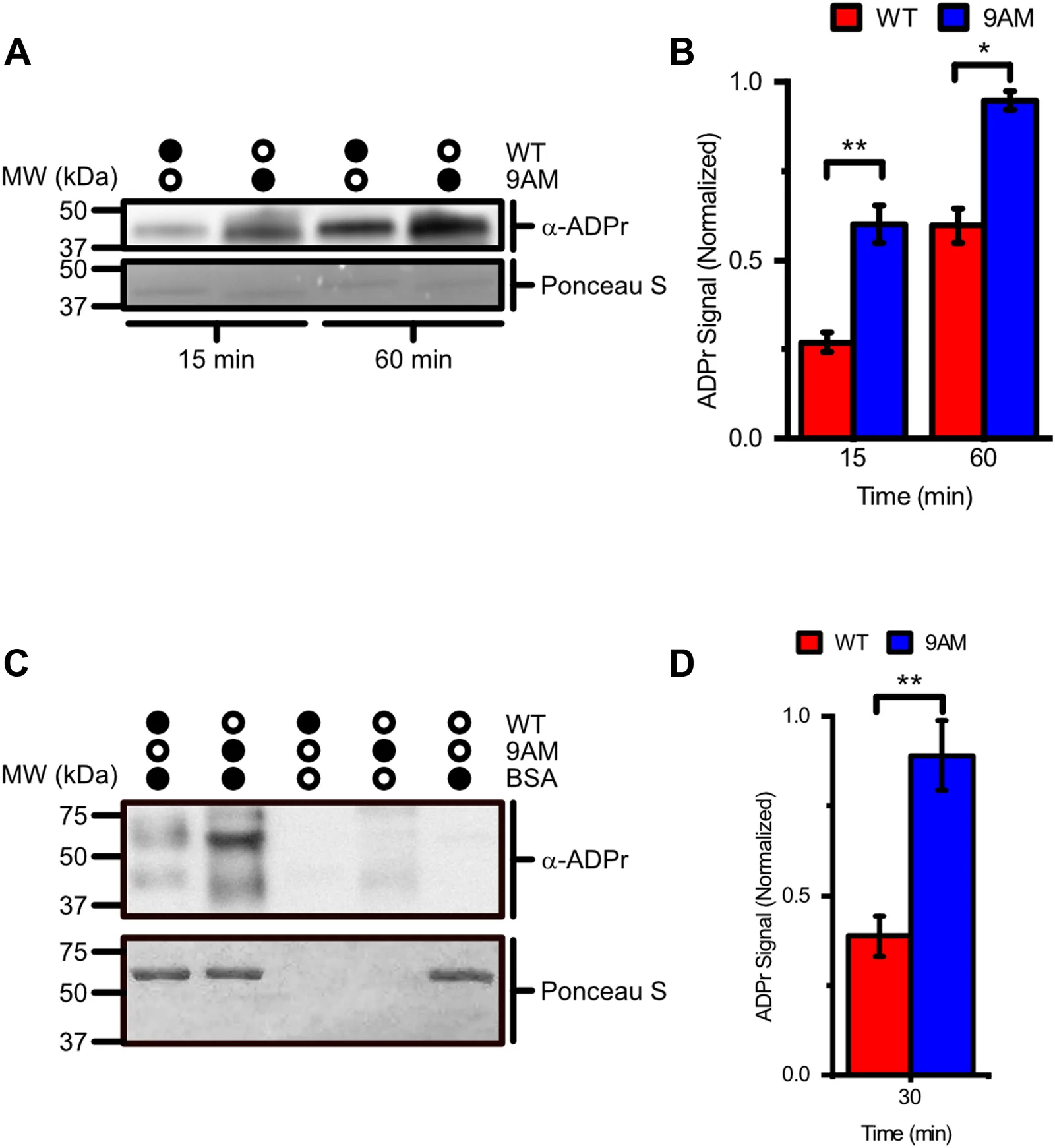
**Validation of MAR sites from the MS/MS screen reveals negative autoregulation.***A*, *in vitro* ADPr assay with WT-PARP14c or 9 AM-PARP14c in the presence of NAD^+^. The same membrane was first imaged with a Ponceau S stain (*bottom image*) and then immunoblotted with AbD33204 to detect substrate ADPr (*top image*). *B*, quantification of results shown in (*A*). The bar graph depicts the auto-ADPr activity of PARP14c at 15 and 60 min (mean ± S.E.M., n = 3). ∗ represents *p*-value <0.05, ∗∗ represents *p*-value <0.01, two tailed Student’s *t* test. *c*, *in vitro* ADPr assay with WT-PARP14c or 9 AM-PARP14c using BSA as a nonspecific substrate in the presence of NAD^+^. The same membrane was first imaged with a Ponceau S stain (*bottom image*) and then immunoblotted with AbD33204 to detect substrate ADPr (*top image*). *D*, quantification of results shown in (*C*). The bar graph depicts the ADPr activity for BSA with PARP14c at 30 min (mean ± S.E.M., n = 3). ∗∗ represents *p*-value <0.01, two tailed Student’s *t* test. ADPr, ADP-ribose; BSA, bovine serum albumin; MAR, mono-ADP-ribose.

As the sites we identified fall amongst three distinct domains, we next wanted to determine how the loss of MAR on these distinct segments would impact PARP14c activity. We generated three separate PARP14c variants where either the 5 MAR sites that form a patch in the KH domain (Pa), the two sites found on the elongated KH helix (KHH), or the two sites in the hinge region (Hi) were substituted for alanine, and we tested their *in vitro* automodification activity against the WT and 9 AM variants of PARP14c (Fig. 5*A*). At both short and long time intervals, the Pa mutant was the most active variant (Fig. 5*B*). As Pa was ADP-ribosylated 8.7-fold and 2.6-fold more (at 15 min and 60 min, respectively) than WT, it seems that these five sites are responsible for the negative regulation of PARP14c activity we observed (Fig. 5*C*). Interestingly, the Pa variant is hyper-active and fully saturates with MAR within 15 min and there is no further increase in modification at 1 h; which is a phenomenon we did not observe with either the WT or 9 AM variants. At 15 min, both the KHH and Hi mutants showed decreased levels of MAR compared to WT. However, this observed decrease was not significant. The deficit in KHH and Hi activity is lost after an hour, which suggests that alternative sites will compensate for the loss of MAR over a longer time period and it might explain why the 15-min result was non-significant.

**Fig. 5:**
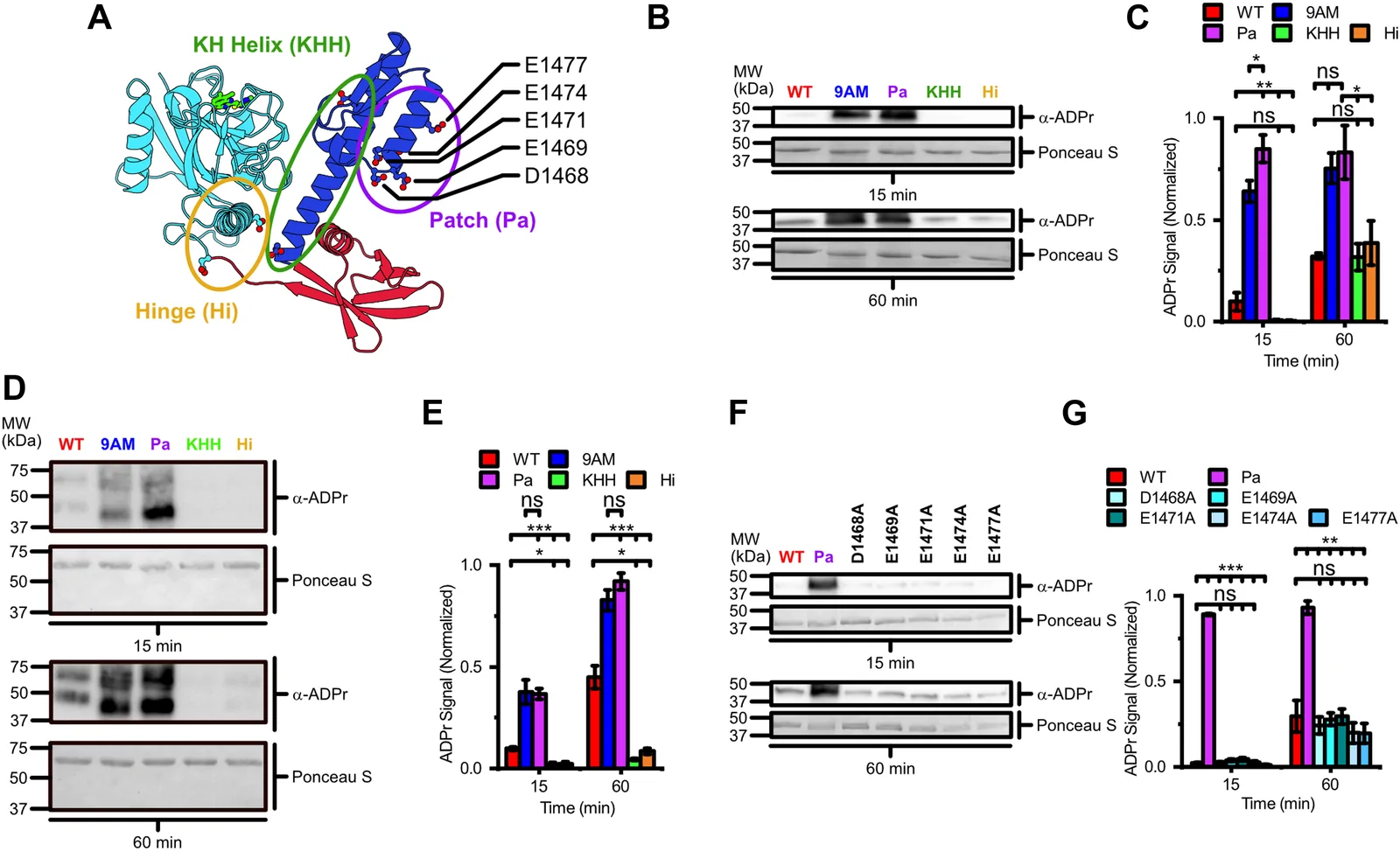
**A cluster of five Asp/Glu residues negatively regulate PARP14 activity.***A*, three distinct clusters of acidic sites were targeted for mutagenesis; the patch (Pa, *purple*), the KHH, (*neon green*), and the Hi, (*orange*). The individual residues in the Pa cluster are indicated. *B*, *In vitro* ADPr assay with WT-, 9 AM-, Pa-, KHH-, or Hi-PARP14c in the presence of NAD^+^. The same membrane was first imaged with a Ponceau S stain (*bottom image*) and then immunoblotted with AbD33204 to detect substrate ADPr (*top image*). *C*, quantification of results shown in (*B*). The bar graph depicts the auto-ADPr activity of the PARP14c variants at 15 and 60 min (mean ± S.E.M., n = 3). *D*, *in vitro* ADPr assay with WT-, 9AM-, Pa-, KHH-, or Hi-PARP14c using BSA as a nonspecific substrate in the presence of NAD^+^. The same membrane was first imaged with a Ponceau S stain (*bottom image*) and then immunoblotted with AbD33204 to detect substrate ADPr (*top image*). *E*, quantification of results shown in (*D*). The bar graph depicts the ADPr activity for BSA with PARP14c at 15 min and 60 min (mean ± S.E.M., n = 3). *F*, *in vitro* ADPr assay with each of the individual alanine point mutants from the Pa cluster. The same membrane was first imaged with a Ponceau S stain (*bottom image*) and then immunoblotted with AbD33204 to detect substrate ADPr (*top image*). *G*, quantification of results shown in (*F*). The bar graph depicts the auto- ADPr activity of the Pa point mutants at 15 and 60 min (mean ± S.E.M., n = 3). ∗ represents *p*-value <0.05, ∗∗ represents *p*-value <0.01, ∗∗∗ represent *p*-value <0.001 two tailed Student’s *t* test. n.s. represents a nonsignificant difference. ADPr, ADP-ribose; BSA, bovine serum albumin; Hi, hinge region; KHH, KH helix.

To determine whether the KHH and Hi sites are potentially activating PARP14c we tested their ability to modify BSA. If these sites activate PARP14c, then their mutation to Ala should result in less MAR signal on BSA; which would be independent of their preferential MARylation by PARP14c on itself. The KHH and Hi mutants display significantly reduced activity with BSA as a non-specific substrate as compared to WT PARP14c (Fig. 5, *D* and *E*). Unlike the results seen with automodification, MAR signal is significantly reduced at both short and long time intervals. These data suggest that the presence of alternative sites on KHH/Hi does not compensate for the loss of PARP14c activity. While we cannot rule out the preference of PARP14c for sites in the KHH and Hi regions, these results are consistent with an activating role for the sites in the KHH and Hi segments. Taken together, our data highlight three distinct regions of PARP14c auto-MARylation that play differential roles in the regulation of PARP14c activity.

Given the conservation of PARP14 across many metazoans (104), we investigated whether the nine PARP14 automodification sites we identified herein are conserved across representative species. We hypothesized that a high degree of conservation would highlight functionally indispensable sites central to PARP14 regulation. Building on prior ortholog identification of PARP14 across primates and metazoans (69, 104), we analyzed amino acid variability at each automodification site across primates and the broader category of metazoans (Supplemental Fig. S1). Within the primates, these sites are nearly invariant, with only E1474 displaying any variation in macaques and gibbons. Across broader metazoans, the Pa sites exhibit a high degree of conservation (with the notable exception of E1474), whereas the KH and Hi sites show marked divergence. Consequently, these data prioritize the Pa sites as primary candidates for universal PARP14 regulation across species, while suggesting that KH and Hi site automodifications play a specialized role fine-tuning function in higher primates.

Based on the negative regulation observed with the Pa variant, we next wanted to interrogate the sufficiency of any single site to drive feedback inhibition. We replaced each of the acidic residues in the patch (D1468, E1469, E1471, E1474, and E1477) with Ala and compared their automodification activity with WT and Pa (Fig. 5, *A* and *F*). None of the individual sites on their own were able to replicate the negative regulation observed with Pa. There was a slight increase in activity with both E1469A and E1471A, and a slight decrease in activity with E1477A at 15 min (Fig. 5*G*), but even the most active single mutant was still 20-fold less active than the Pa variant. After an hour, all of the single-point mutants recapitulated the activity of WT PARP14c. These data collectively indicate that the negative regulation we observe is more complex than initially thought and may require the modification at multiple sites to achieve. Though, how many are required or the level of potential redundancy in this area remains unclear.

## Discussion

The enzymes that comprise the PARP family are increasingly linked to a host of cellular functions and disease outcomes (116). Like other PTMs, ADPr itself represents a way for the cell to dynamically alter the structure and function of the targeted biomolecules. In order to fully understand how individual ADPr modifications on specific targets drive downstream effects, there is an on-going need for proteomic methods to identify and validate ADPr sites. A noted complication with confident site assignment is the fragility of ADPr under the collisional forces used in most MS/MS workflows–which primarily rely on HCD. ETD has emerged as an alternative for site identification, but this method carries additional costs and technical challenges that decrease its overall accessibility. Herein, we optimized an ADPr MS workflow that is entirely compatible with HCD, can be completed in a day, carries a low cost, and is accessible to any standard proteomics resource. Importantly, the incorporation of a stable HA-tag allows for high pH fractionation during LC-MS analysis which increases the depth of sequencing over other methods that require a low pH to stabilize the ADPr ester. Our method improved the true-positive site identification rate by nearly two-fold and allowed us to validate the identified sites *in vitro*.

As with any proteomics optimization strategy, it is essential to consider the limitations of the method and the remaining concerns that should be addressed in future work. Firstly, as our primary goal in the described work was to optimize an MS method for the detection of acidic ADPr linkages, we purposefully limited our study to *in vitro* site identification to allow us to rapidly assess the impacts of incubation conditions on true positive site discovery. It is important to note that this choice introduces a level of artifice that does not fully recapitulate the conditions PARP14 experiences *in vivo*. This strategy allowed us to identify some of the fundamental parameters that govern HA attack, but it necessarily removes the greater context found in a cell. As a logical next step, it will be important to integrate our optimized conditions in proteomic workflows that are designed to investigate ADPr *in vivo*. Connected to this concern, it will be essential to determine whether the optimized conditions we’ve identified will work with cellular culture or primary tissue samples.

Secondly, as discussed above, it is clear that PARP14 forms protein-protein complexes with various partners (73, 82, 85) (notably, PARP9/DTXL3 (80, 88)) and this interaction might alter its automodification profile. Whether PARP14 automodification, and therefore autoregulation, is dependent on its quaternary structure is an important mechanistic concern. We are interested in leveraging our optimized conditions to address this question in future proteomics efforts with PARP14 interactors (potentially including DNA and RNA (73)).

Thirdly, while we purposefully chose BSA as a nonphysiological target for PARP14c *trans* modification, it will be important to assay the automodification mutants identified in this study with additional physiological targets of PARP14 (*e.g*. PARP13 (93), RACK1 (85), XRCC1 (41), RPA (41), etc.). This will allow us to determine how autoregulation might impact *trans* MARylation by PARP14.

Fourthly, while we purposefully chose a truncated form of PARP14 to limit the complications caused by its many additional N-terminal domains as we optimized HA incubation conditions, it will be vital to reintroduce these domains into our pipeline in future work. With the improvements described herein, it will be interesting to deploy our optimized pipeline with variants of PARP14 that retain either MAR reading and/or erasing activities to discern how these functions alter automodification. We are interested in determining how each of PARP14’s domains impacts its automodification profile and the effects of each putative ADPr site within the context of the full-length construct.

As a final caveat, we cannot rule out the possibility that the autoregulatory effects we observed for PARP14c were not solely due to lost ADPr sites, but due to the overall loss of negative charge caused by the mutation of Asp/Glu to Ala. It is possible that the mutation of nine acidic residues (or even as few as two) might substantially impact the overall charge and structure of the protein. While retained catalytic activity with each of the PARP14c variants suggests that the core of the catalytic domain remained grossly intact, it will be interesting to assess how the loss of either MAR sites or acidic “patches” (or a combination of the two) alters PARP14 structure and function. Even with these caveats in mind, our efforts provide an important entry point towards uncovering the PARP14 autoregulatory mechanism.

A complicating factor with site identification is the recent discovery of ADPr attachment at a variety of amino acids (49)–arginine, lysine, cysteine, serine, His, tyrosine, and the aforementioned Asp/Glu. These acceptor sites create distinct bonds with the ribosyl sugar, which will have differential chemical outcomes in the cell and MS/MS workflows (62). Recent work in our lab and others has highlighted the unique challenges with acidic linkages in particular; notably, they have a very short half-life even in mild conditions (54, 55, 56). As such, it is highly likely that previous proteomic efforts were underrepresenting the number of acidic site modifications. Conversion of MAR esters to an HA moiety replaces a labile PTM with a stable chemical signature, which overcomes this issue with ADPr hydrolysis during sample prep, and furthermore introduces a stable modification that is preserved during HCD fragmentation. However, even though HA labeling has been deployed previously in MS/MS efforts, it was completed in mild conditions with several hours-long incubation steps. Using our TLC-MALDI technique, we demonstrate that under those conditions there is a substantial loss of ADPr to hydrolysis without the requisite HA tagging. By lowering the pH and increasing the temperature slightly, we were able to increase the level of successful HA tagging by approximately two-fold. We also show that this step is completed in an hour, lowering the incubation time substantially from previous efforts. In sum, it is clear that care need be taken when designing MS/MS pipelines to identify ADPr sites and we have developed a simple method to increase *bona fide* acidic site identifications. This method has the potential to identify the targets of other Asp/Glu-specific PARPs, and determine what effects overlooked sites have on cellular biology.

An additional complicating factor when attempting to address PARP family-member specific function is the observation that many of the PARP family members (including PARP14) are known to form complexes in the cell (93, 117, 118). As such, it can be quite difficult to ascertain whether any endogenously observed ADPr site identified from cellular extracts arose from an automodification event or was catalyzed in *trans* by a different PARP family member (119). While several recent proteomics efforts have identified MAR sites on PARP14 (Supplemental Table S5), this key detail regarding *cis versus trans* labeling has been lacking. By utilizing only PARP14c *in vitro*, and validating these putative automodification sites, our work provides an important advance in addressing this outstanding mechanistic question.

As previously mentioned, an interaction between PARP14 and the PARP9/DTX3L complex was recently identified (80, 88), which results in the down-regulation of PARP14 activity and expression via PARP9/DTX3L. Kar and colleagues demonstrate that DTX3L and PARP14 interact primarily through the KH8 domain of PARP14 and the KH2 domain of DTX3L. They further show that mutation of a series of seven residues on the KH8 domain of PARP14 results in a loss of DTX3L binding and, therefore, a relief from DTX3L-mediated repression. Interestingly, among the mutations they studied were two of the sites (D1468 and E1469) we identified from our screen in the Pa region (80). In their experiments–with the same truncated form of PARP14 that we employed–they do not observe an increase in PARP14 automodification upon the loss of D1468/E1469, which agrees with our observation that multiple sites from Pa are able to repress PARP14 activity. Combined with our results, these data suggest that the Pa region is important for multiple types of negative regulation, both with PARP14 itself and the PARP9/DTX3L complex. Further work will be required to determine how intra- and intermolecular regulation of PARP14 is carried out in the cell, but our efforts should aid researchers in deciphering this mechanism with the newly identified sites, and an improved experimental framework for studying the sites of other PARPs.

A long-standing question in the PARP field is whether MAR site selection is specific. Outside of a general–and not necessarily family-wide–preference for a lysine residue N-terminally adjacent to the modification site (94, 108, 120), there is scant evidence for an ADPr motif. While a few ADPr sites have been shown to alter enzyme function (121), they are dwarfed by the thousands of proposed sites from MS/MS efforts. Therefore, whether MAR is attached to specific sites, driving specific alterations in protein function, or whether any (or some) of the solvent accessible sites are sufficient to trigger downstream consequences will have a significant impact on ADPr-dependent biology. In the case of acidic sites, this issue is further complicated by the observation that the esters formed with ADPr are known to reorganize on the ribosyl ring (55, 122). It has been suggested that the opening of the ring during this isomerization would provide a non-enzymatic mechanism for site transfer between neighboring acceptor residues on target proteins. Whether this intramolecular transfer is physiologically relevant is also an open question. Our work has identified a set of residues on PARP14c that collectively down-regulate the activity of PARP14 itself. While we have demonstrated that no single residue is capable of driving this negative autoregulation, future work will be required to disentangle the effect of this patch on PARP14 function. Whether there is a threshold of ADPr within this patch required to turn down PARP14 activity–akin to the regulatory behavior seen with the phosphorylation of the C-terminal domain of RNA Polymerase II (123)–or whether a single or multiple site(s) within this region are sufficient, is an essential future concern as we continue to study this autoregulatory mechanism. Further, whether this region gets modified once–and then passes the MAR around the acceptor residues–will require additional biochemical and structural investigation beyond the scope of the current work.

At the same time, our work revealed two distinct sets of two residues each that collectively up-regulate the activity of PARP14c. Unlike the Pa region, these residues in the KH and Hi constructs appear to play a minimal role in regulating automodification, yet they clearly increase the ability of PARP14c to modify BSA in *trans*. Alternatively, it is also possible that instead of activating PARP14c via automodification, these sites play a more nuanced role in promoting *trans*-modification. Both the specificity of this activation by the KH and Hi residues, and the mechanism of activation mediated by these four sites, are ongoing research questions. More broadly, the distinct conservation patterns of the Pa, KH, and Hi sites suggest lineage-specific functional roles outside of humans. This natural sequence variation provides a valuable framework for engineering chimeric PARP14 constructs as investigative tools. Ultimately, how these regulatory sites evolved in response to divergent selective pressures–like viral infections–remains an intriguing open question for future investigation. Collectively, our current data provide an important entry into addressing the specificity of ADPr modification and the mechanisms that link MAR modification to enzyme function.

As with many other PTM families, the removal of ADPr is a core aspect of its function in the cell. Just like the PARP family itself, there are outstanding questions in the field regarding the specificity of the glycohydrolases that remove ADPr from target macromolecules. Do these “erasers” work on all ADPr sites with the same specificity or do they also display a preference for certain sites? Further, is this preference dictated by solvent accessibility of the modified amino acid, the ability of the ribose ring to rearrange or linearize on certain residues and not others, or additional structural parameters? Previous work in our lab using ADPr-peptides demonstrated a difference in glycohydrolase activity based on the primary sequence of the ADP-ribosylated motif (54). However, how these preferences might be leveraged towards selective ADPr removal on a full protein or in the cell remains unclear. Both the PARP14 mutants that we have developed in the current study, and the optimized methods for ADPr site identification, could provide important information on macrodomain specificity and allow us to make progress on addressing this open question.

As previously mentioned, PARP14 plays important roles in the innate immune response to various viral infections (74, 79). The misregulation of PARP14 can lead to overexpression and an upregulation of activity that has been linked to a variety of cancers–notably, multiple myeloma (29, 124), hepatocellular carcinoma (28), and the reprogramming of tumor-associated macrophages (31). Our data has validated a set of residues that might play a role in both stimulating PARP14 activity during infection (sites in the KHH and Hi region) and that are required to prevent the hyperactivation of PARP14 in an oncogenic context (sites in the Pa region). It will be interesting to determine how these sites impact both the physiological roles of PARP14 and its impact in cancer cell development. We look forward to the incorporation of the MAR sites variants we have identified in future investigations of PARP14 function *in vivo* and the utility of our MS/MS pipeline in identifying putative PARP14 sites in these various contexts. Finally, we believe that the constitutively active form of PARP14c that we have developed will be immediately useful in drug screening efforts and *in vitro* assays where PARP14 is being deployed.

## Data Availability

The mass spectrometry proteomics data have been deposited to the ProteomeXchange Consortium (129) via the PRIDE (130) partner repository with the dataset identifier PXD076709 and 10.6019/PXD076709.

## Supplemental Data

This article contains supplemental data (56, 60, 81, 109, 113, 114).

## Conflict of Interest

The authors declare no competing interests.
