# Supplementary material for: Optimized Detection of Acidic ADP-Ribosylation by PARP14 in Vitro Using Multi-Modal Mass Spectrometry Reveals Putative Autoregulatory Patches
Source: Mol Cell Proteomics. 2026 Sep 21;25(10):101666. doi: 10.1016/j.mcpro.2026.101666 (PMC13694955; doi:10.1016/j.mcpro.2026.101666)
Supplement: Supplementary file 1 [file mmc1.docx]

Ian Carter-O’Connell
Santa Clara University
Department of Chemistry and Biochemistry
500 El Camino Real
Santa Clara, CA 95053, United States
Phone: 408-554-5033
E-mail: [icarteroconnell@scu.edu](mailto:icarteroconnell@scu.edu)

**Optimized detection of acidic ADP-ribosylation by PARP14 *in vitro* using multi-modal mass spectrometry reveals putative autoregulatory patches**

Steph Nivaggioli^†1^, Hannah H Nguyen^†1^, Kiana K Harker^†1^, Christian M Mohr^†1^, Jake Wood^1^, Mikayla Dupen^1^, Zeeshan Javed^1^, Natalia Karjalainen^†1^, Violetta Yousefpor^†1^, Ashok Reddy^2^, and Ian Carter-O’Connell^*1^

^1^Department of Chemistry and Biochemistry, Santa Clara University, Santa Clara, CA, USA
^2^Proteome Shared Resource, Oregon Health and Science University, Portland, OR, USA

^†^Authors contributed equally

^*^Author to whom correspondence should be addressed ([icarteroconnell@scu.edu](mailto:icarteroconnell@scu.edu))

**Figure S1.** Conservation of PARP14c ADP-ribosylation sites across primates and metazoans. The domain structure of PARP14c is shown with domain colors as in Fig 3b. The ADPr sites identified in Fig 3a are indicated by open circles and the residue number is indicated based on the human sequence. Conserved Asp/Glu residues are indicated in bold.

**Figure S2.** Purified proteins utilized in this study. All proteins were loaded at a concentration of 500 µM and visualized using Coomassie stain following separation on an SDS-PAGE gel. The expected recombinant protein for each sample is indicated by an *. The concentrations of PARP14c were then adjusted based on their normalized pixel intensity from this gel prior to incubation in further activity assays.

**Figure S3.** Unaltered and uncropped scans of Western blots and gels supporting the data presented in this manuscript. Molecular weights (kDa) are indicated for each blot and/or gel. The cropped regions presented in the manuscript are indicated by a bound box. The three replicates used for each experiment are indicated by their respective trial numbers. All lanes for any individual experiment originate from the same exposed blot. (**a**) Uncropped blot corresponding to the anti-ADPr immunoblot shown in Figure 4a. (**b**) Uncropped blot corresponding to the Ponceau S-stained blot shown in Figure 4a. (**c**) Uncropped blot corresponding to the anti-ADPr immunoblot shown in Figure 4b. (**d**) Uncropped blot corresponding to the Ponceau S-stained blot shown in Figure 4b. (**e**) Uncropped blot corresponding to the anti-ADPr immunoblot shown in Figure 5b. (**f**) Uncropped blot corresponding to the Ponceau S-stained blot shown in Figure 5b. (**g**) Uncropped blot corresponding to the anti-ADPr immunoblot shown in Figure S1a. (**h**) Uncropped blot corresponding to the Ponceau S-stained blot shown in Figure S1a. (**i**) Uncropped blot corresponding to the anti-ADPr immunoblot shown in Figure 5d. (**j**) Uncropped blot corresponding to the Ponceau S-stained blot shown in Figure 5d.
